# Primary total hip arthroplasty for a femoral neck fracture in a homolateral above-knee amputee: a case report

**DOI:** 10.3389/fsurg.2025.1732504

**Published:** 2026-01-08

**Authors:** Changli Xu, Yu Yin, Hongchen Shi, Zhengqiang Li, Suchi Qiao, Jianli Bu

**Affiliations:** 1 Department of Orthopedics, Bethune International Peace Hospital (The 980th Hospital of the People's Liberation Army Joint Logistics Support Force), Shijiazhuang, China; 2 Department of Orthopedics, The Third Affiliated Hospital of Naval Medical University, Shanghai, China

**Keywords:** above-knee amputations, femoral neck, hip fracture, total hip arthroplasty, total hip replacement

## Abstract

**Introduction:**

Femoral neck fracture in a homolateral amputated extremity is not common and challenging for the surgeon to manage. In this article we share our experience of this unusual entity.

**Methods:**

We present a case of a displaced femoral neck fracture of the right femur in a 69-year-old female, who underwent at the age of 29 an above-knee amputation of the homolateral limb. The fracture was managed by a primary total hip arthroplasty (THA). The post-operative course was uneventful. There was no infection, deep vein thrombosis, dislocation, or any other complication.

**Results:**

Over one-year follow-up demonstrated that the patient while wearing the prosthetic limb received satisfactory and functional use of normal activities. The patient achieved a good functional outcome with a Harris Hip Score at 86/100. To date, the patient has returned to normal activities without symptoms.

**Conclusion:**

Treating femoral neck fractures in homolateral above-Knee amputees is challenging. Surgical technical tips and rehabilitation exercises are necessary and crucial. Total hip arthroplasty can provide satisfactory functional outcome and return to pre-fracture daily life activities.

## Introduction

Femoral neck fractures are a common source of morbidity and mortality worldwide, which account for approximately 3.6% of all fractures (1). Nevertheless, the discovery and development of hip arthroplasty have improved its prognosis, with a high survival rate, and satisfactory functional results. Although total hip replacement has been well described in the literature especially for healthy individuals, femoral neck fracture in an amputated extremity is not common and little has been published about this condition (2). There is little information about the technical features and outcomes of the surgery for lower limb amputees. Femoral neck fracture on amputated limb is an uncommon lesion and challenging for the surgeon to manage because of technical difficulties such as the patient setup, the surgical approach, immobilization, surgical reduction, and stabilization, as well as post operative rehabilitation. The goal of treatment is the return of the amputee to their pre-fracture status that includes satisfactory and functional use of the prosthetic limb.

In this study we present a rare case of femoral neck fracture in a patient with homolateral femoral amputation, treated with a primary total hip arthroplasty. Written informed consent for publication was obtained from the patient, and the case was reviewed by the ethics committee.

## Case report

**Figure 1:**
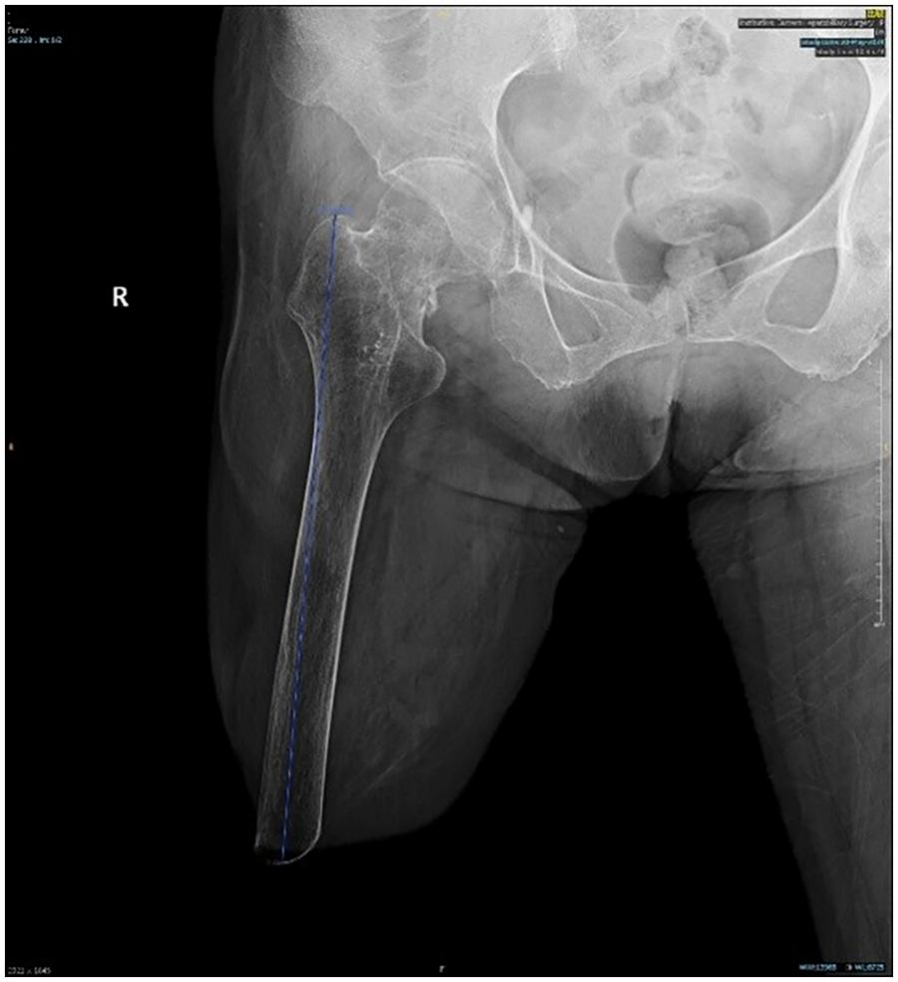
Preoperative antero-posterior radiograph of the pelvis showing a displaced femoral neck fracture.

A 69-year-old female, who underwent at the age of 29 an above-knee amputation of the right higher limb as a life-saving procedure after a traffic accident, was taken to the emergency room for homolateral hip trauma. She tripped and fell down during walking, striking the lateral aspect of her right hip on the ground. She was diagnosed with a grade IV femoral neck fracture, according to Garden's classification (Figure 1). Before this injury, she was previously mobilizing using a prosthesis and returned to her normal activities.

On physical examination, there was tenderness in the greater trochanter and groin area. Right thigh stump showed tenderness on the log roll test, and there was axial percussion pain. The overlying skin was intact, with a well healed surgical scar over the stump and normal sensation and circulation. There were no any vascular or neurologic complications. And no other injuries were identified.

**Figure 2:**
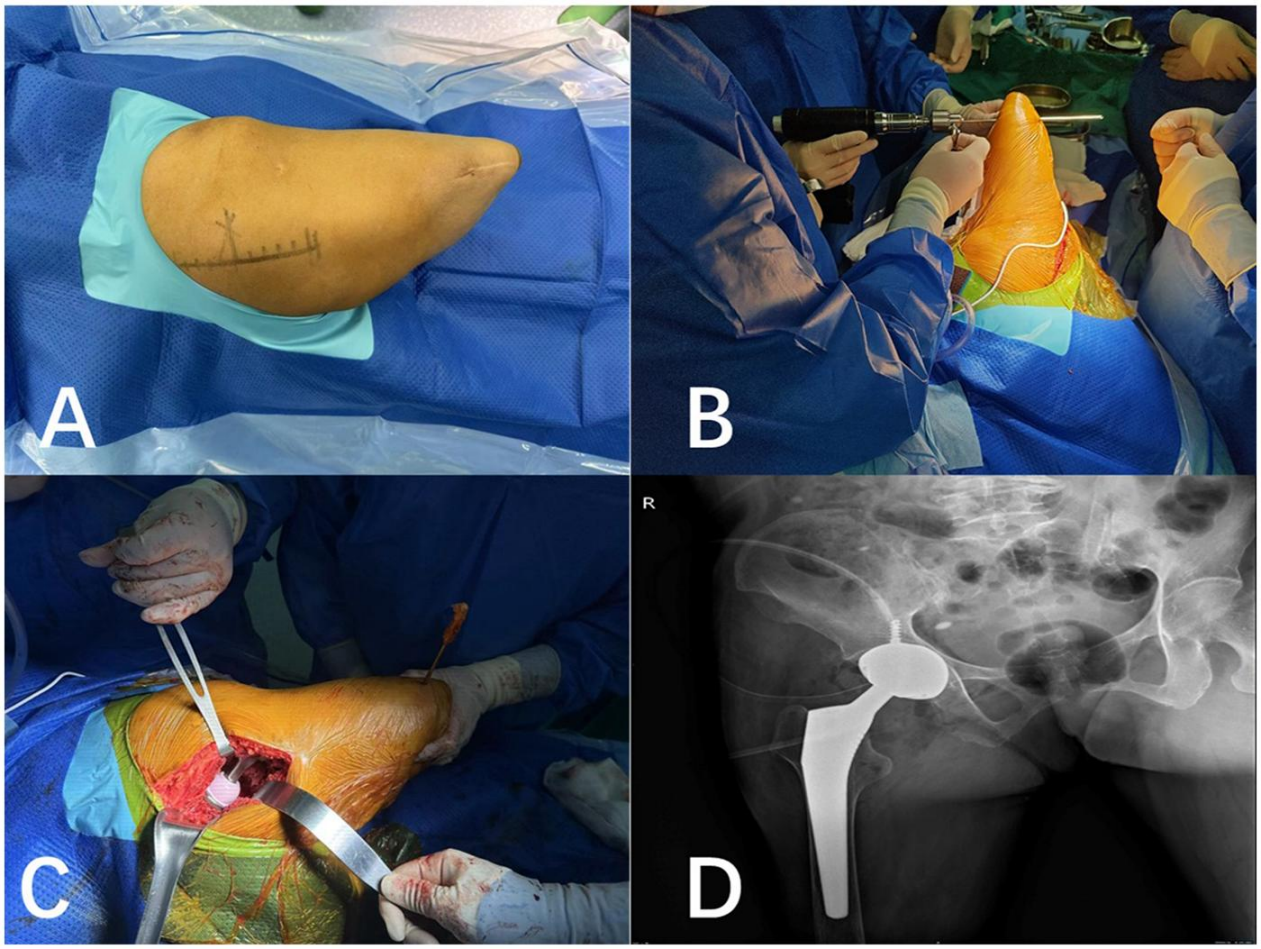
**(A)** the posterior lateral surgical incision of the right hip joint. **(B)**The Steinmann pin inserted in the distal femur. **(C)** Intraoperative image showing the THA for the patient. **(D)** Postoperative radiographs revealed satisfactory.

Considering the patient's age and the need for rapid recovery, total hip arthroplasty (THA) was more suitable for this patient. The radiograph (Figure 1) included the complete stump which could assess the remaining femoral length and the width of the medullary canal. Figure 1 showed the residual limb length was 176 mm from the greater trochanter to the distal end with significant bone mass loss. Given that the patient has no underlying diseases and a long life expectancy, the cementless implant was a good candidate for this patient. However, further care should be taken to the implanting of prothesis to avoid intraoperative fracture. Following informed consent, the procedure was performed, under general anesthesia, with the patient in a left lateral decubitus position. A posterior lateral surgical approach of the right hip joint, with an approximately 12 cm skin incision centered around the greater trochanter of the femur, was required (Figure 2A). A Steinmann pin was inserted in the distal femur to facilitate the control of the stump (Figure 2B). A 48 mm porous coated uncemented acetabular shell (IRNENE Co., China) was aligned with the transverse acetabular ligament. The acetabular shell was transfixed with two 6.5 mm cancellous screws (IRNENE Co., China). A cementless size 15 type femoral stem was inserted (IRNENE Co., China) and a 32 mm Ceramic liner was used (IRNENE Co., China). Intraoperative testing confirmed stable mobility of the hip joint (Figure 2C).

**Figure 3:**
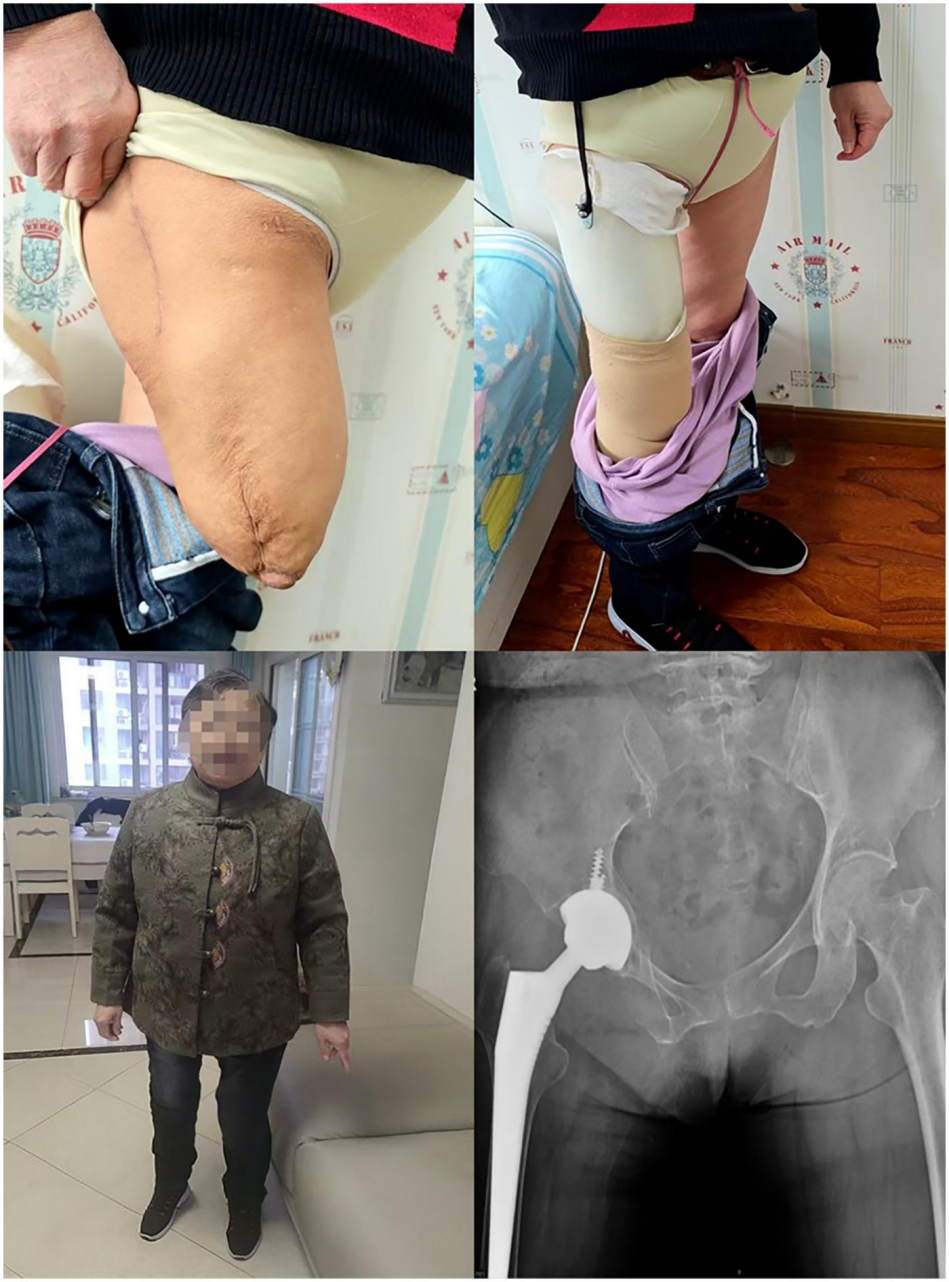
Postoperative radiographs and clinical pictures with prosthesis at 1 year-follow-up.

**Figure 4:**
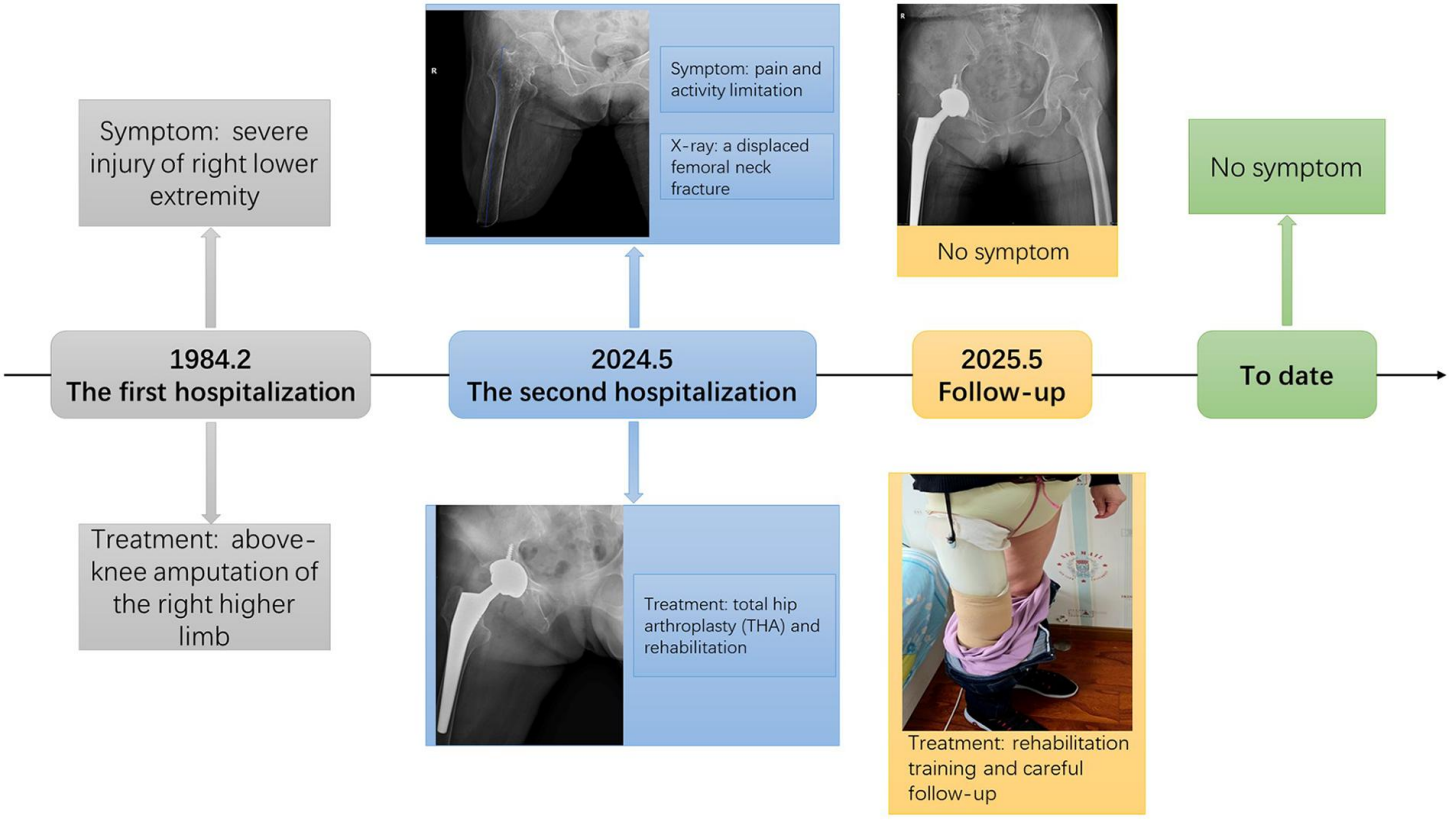
The timeline for this patient.

Post-operatively, antibio prophylaxis for 72 h as well as sub cutaneous anticoagulation was prescribed to prevent thrombo-embolic and infectious. The post-operative course was uneventful. There was no infection, deep vein thrombosis, dislocation, or any other complication. We treated osteoporosis with zoledronic acid, calcium and vitamin D3 perioperatively. During her hospitalization, she was allowed to walk with a walking aid and underwent physiotherapy. She was discharged home five days after surgery. Two weeks postoperatively the wound had healed. Utilizing the assistance of a walker, she embarked on wearing prosthetics for her rehabilitation program, encompassing gait training and endurance training. Another two weeks later, she began to walk normally wearing her prosthesis without any walking aid or crutch. At the most recent follow-up, one year following THA, the amputation limb exhibited satisfactory condition (Figure 2D). Upon examination, the patient demonstrated good range of motion in the hip joint without any pain: her hip flexion was 90°, external and internal rotation was 30°, abduction was 40° and adduction was 20°. While wearing the prosthetic limb, no discrepancy in leg length was noted. The functional outcome was good with a Harris Hips Score at 86/100. The x-ray images revealed that the femur and acetabular components were well-incorporated, with no signs of osteolysis. To date, the patient has returned to normal activities without symptoms (Figure 3). Figure 4 shows the timeline of the complete illness course of this patient.

## Discussion

This 69 years old female, active, trans-femoral amputee underwent cementless THA without complications and achieved her preoperative activity status, wearing her artificial limb prosthesis. However, residual lower limb fracture is not common, with little information available in the previous literature. Long-term wearing of prosthetics may induce osteo-articular as well as muscular modifications in residual lower limb. Total hip arthroplasty in amputees can be very challenging, with serious technical issues such as surgical preparation, hip dislocation and reduction, component positioning and rehabilitation. Therefore, a detailed plan is necessary for this surgery.

We conducted a systematic literature search on PubMed and Google Scholar for case reports published between 2009 and 2025 (Table 1). The keywords used included above-Knee amputation, total hip arthroplasty and femoral neck fracture.

**Table 1:** Summary of reported cases of femoral neck fractures in patients with ipsilateral above-knee amputations.

Author/year	Patient age/sex	Age/cause of amputation	Cause/type of femoral fracture	Surgical data	Reported outcomes
Tripathy et al. 2023 (15)	20/M	20/traffic accident	Traffic accident/garden Ⅳ type	Posterior approach Uncemented prothesis One 4.5 mm Steinman pin inserted into the remnant of femur 3 cm proximal to the amputation site	10 months follow-up: walk with prosthetic limb without pain or any sign of osteolysis or subsidence
Milella et al. 2024 (16)	85/M	83/obliterating vasculopathy	Fall/garden Ⅳ type	Direct lateral approach Uncemented prothesis No instruments inserted in anatomic landmarks	6 months follow-up: walk with prosthetic limb without pain
Christidis et al. 2022 (14)	61/M	60/	Trauma/garden III type	Lateral (Hardinge's) approach Cemented prosthesis No instruments inserted in anatomic landmarks	6 months follow-up: walk with prosthetic limb without pain
Ma et al. 2015 (17)	67/M	30/trauma	Fall/garden III type	Posterior approach Uncemented prothesis One Steinmann pin inserted at the greater trochanter	2 years follow-up: walk with prosthetic limb without pain or limitation
Zhao et al. 2025 (9)	64/M	34/work-related injury	Fall/garden III type	Direct anterior approach Uncemented prothesis and bone cement applied at the distal end of the femoral canal Wires wrapped around the femoral shaft of the residual femur	6 months follow-up: walk with prosthetic limb without pain and 84.78 of the Harris Hip Score
Gruhonjic et al. 2022 (18)	89/M	teenager/firearm injury	Fall/garden III type	The Femoral Neck System (FNS) One traction pin inserted in the distal femoral shaft	1 year follow-up: walk with prosthetic limb without pain
Perumal et al. 2017 (6)	75/M	75/traffic accident	Traffic accident/garden III type	Lateral approach Cemented bipolar hemiarthroplasty two 6.5 mm Schanz pins applied perpendicular to each other with one pin in anteroposterior plane and the second pin in sagittal plane	14 months follow-up: walk with prosthetic limb without pain
Kandel et al. 2009 (8)	68/M	10/firearm injury	Fall/garden III type	Posterior approach Uncemented bipolar hemiarthroplasty A bone holder used in the subtrochanteric area	5 years follow-up: walk with prosthetic limb with normal gait and no pain

According to literature reports, for THA after lower extremity amputation, the posterior surgical approach was used for 51.2%, anterolateral approach for 48.2% and direct anterior approach for 0.6% (3). The selection of surgical approach does not significantly influence surgical outcomes and is predominantly determined by the surgeon's personal preference. The majority of surgeons prefer the posterior approach, as it facilitates the exposure of the hip joint and the process of joint replacement.

There are some difficulties during the surgical process for above-knee amputees. The short lever arm with difficulties to handle the proximal femur during the hip dislocation and implants insertion. Some surgeons inserted a Steinman pin into the great trochanter, while someone inserted Schanz pins into the distal femur, to provide rotational control and facilitate dislocation/relocation (4–6). Some surgeons resorted to use bone clamps or hooks in the intertrochanteric region, while someone used bone clamps or forceps on the proximal femoral shaft itself, up to 5 cm distal to the lesser trochanter (7–10). Meanwhile, positioning the femoral stem within the femoral canal, regarding anteversion, poses a significant challenge in such procedures in above knee amputees. It can be achieved by palpating the lesser trochanter and following the anatomic alignment of the femoral canal, during its preparation for insertion of the uncemented femoral prosthesis. In some cases, several technical tips may be necessary for patients with above-knee amputation. The contracture of the hip flexors and abductors may require soft tissue release. The femoral stem should be placed with adequate length and in the appropriate depth according to the level of amputation. Of note, a larger femoral head is preferred to be selected to ensure better stability. Considering that the muscle tone of patients with femoral amputation is weaker than that of normal individuals, we made the head socket slightly tighter during the surgery to reduce postoperative dislocation. Additional care should be brought to the skin of residual limb during closure of the operative wound to protect the scar subsequently from pressure related to the limb prosthesis. Compression wraps and bandages could be routinely utilized in amputees to prevent this occurring.

Another important factor to be taken into consideration is osteoporosis caused by the amputation which can lead to preoperative or post-operative peri prosthetic fractures, or an early loosening of the implant. Pre-operative planning is extremely important; radiographs or CT scans should include the full-length of the remaining femoral to assess the bone mineral density and distal bony geometry and facilitate preoperative planning for prosthesis selection (11). If patients with osteoporosis, there is an increased risk of intraoperative calcar fracture during femoral prosthesis implantation. To mitigate this risk, some authors suggest using cerclage wires preoperatively to reduce calcar fracture incidence. In those patients, the use of cemented implants should be considered. If patients don't have a diagnosis of severe osteoporosis and have a long-life expectancy, the cementless prosthesis is a good choice, given long-term risks of cement aging and late loosening (3, 9, 12). And muscular deficiency in the residual limb increases the risk of hip dislocation (13, 14). Hence, it is of utmost importance to initiate rehabilitation exercises at the earliest possible opportunity.

## Conclusion

Treating femoral neck fractures in homolateral above-Knee amputees can be challenging. Total hip arthroplasty provides a better rehabilitation with an early full-weight bearing and return to ambulation in the management of femoral neck fractures. Surgical technical tips and rehabilitation exercises are necessary and crucial. Satisfactory functional outcome and return to pre-fracture daily life activities can be achieved after THA.

## Data Availability

The original contributions presented in the study are included in the article/Supplementary Material, further inquiries can be directed to the corresponding authors.
